# Machine learning-based prediction of 6-month functional recovery in hypertensive cerebral hemorrhage: insights from XGBoost and SHAP analysis

**DOI:** 10.3389/fneur.2025.1608341

**Published:** 2025-06-04

**Authors:** Menghui He, Zhongsheng Lu, Yiwei Lv, Zihai Cheng, Qiang Zhang, Xiaoqing Jin, Pei Han

**Affiliations:** ^1^Department of Graduate School, Qinghai University, Xining, China; ^2^Department of Neurosurgery, Qinghai Provincial People's Hospital, Xining, China

**Keywords:** hypertensive cerebral hemorrhage, predictive model, XGBoost, SHAP, machine learning

## Abstract

**Background:**

The poor prognosis of hypertensive cerebral hemorrhage (HICH) remains high. The period of 3–6 months after onset is the most rapid phase of neurological recovery in hemorrhagic stroke patients. Accurate early prediction of 6-month functional outcomes is critical for optimizing therapeutic strategies. This study compared the predictive efficacy of multiple machine learning models to identify the optimal model for forecasting long-term prognosis in HICH patients.

**Methods:**

We conducted a retrospective analysis of clinical data from 807 HICH patients admitted to Qinghai Provincial People's Hospital's Neurosurgery Department between June 2020 and June 2024. After data preprocessing, data from June 2020 to December 2023 (*n* = 716) were randomly split into training (*n* = 497) and test sets (*n* = 219) at a 7:3 ratio. Data from January to June 2024 (*n* = 91) served as an external validation set. Recursive Feature Elimination (RFE) was performed to identify optimal features, and repeated five-fold cross-validation minimized the risk of overfitting. Model performance was evaluated using Area Under the Curve (AUC) and Decision Curve Analysis (DCA) across XGBoost, Random Forest (RF), Logistic Regression (LR), Support Vector Machine (SVM), and K-Nearest Neighbors (KNN). The optimal model was interpreted via SHapley Additive exPlanations (SHAP).

**Results:**

The 6-month poor prognosis rate among 807 HICH patients was 27.51%. The XGBoost model exhibited optimal performance in the training set (AUC = 0.921, 95% CI: 0.896–0.944) and demonstrated stability in the external validation set (AUC = 0.813, 95% CI: 0.728–0.899). DCA analysis showed that the XGBoost model provided higher net benefit than other models across threshold probabilities of 0%−20% and 56%−100%. SHAP analysis identified hematoma volume as the most critical predictor, with secondary contributions from Glasgow coma score, white blood cell count, age, serum albumin, and systolic blood pressure, among others.

**Conclusion:**

XGBoost models demonstrate powerful accuracy in long-term prognosis prediction of HICH patients. The SHAP framework quantifies the specific contributions of key pathophysiological indicators to individual patient model predictions, enabling individualized risk stratification and strategic allocation of medical resources.

## 1 Introduction

Hypertensive cerebral hemorrhage (HICH), caused by the rupture of small blood vessels due to chronic hypertension, affects approximately 4 million individuals globally each year (1). HICH is associated with high mortality and disability rates, posing a significant threat to patient health and survival (2). As a critical neurosurgical condition, HICH is clinically characterized by acute onset, rapid progression, and associated complications (3, 4). Post-hemorrhagic motor recovery predominantly occurs within the first 3–6 months post-onset (5, 6). Consequently, early prediction of neurological recovery beyond 6 months and development of an effective prognostic system hold substantial clinical value, as they are essential for optimizing medical resource allocation, guiding individualized treatment strategies, and improving functional outcomes in affected patients (7).

Machine learning's powerful data-processing capabilities, adaptability, and proficiency in capturing non-linear patterns render it highly suitable for analyzing multifaceted clinical datasets, with applications in clinical research growing annually (8). Their capacity to analyze large-scale, intricate data makes them indispensable for clinical diagnosis and outcome assessment. SHAP is a strong method in the realm of machine learning interpretability. This method demystifies the “black-box” nature of complex models, thereby enhancing transparency and credibility in model outcomes (9).

This study aimed to compare multiple machine learning models using diverse clinical features to predict long-term prognosis in HICH patients. Following screening, SHAP analysis was performed on the best-performing models. By identifying key clinical metrics that influence prognosis prediction, we aimed to make the decision-making process of the models transparent.

## 2 Methods

### 2.1 Data sources

We retrospectively collected clinical data from 807 patients diagnosed with HICH admitted to the Department of Neurosurgery at Qinghai Provincial People's Hospital between June 2020 and June 2024. These patients were included in the study cohort. Specifically, data from June 2020 to December 2023 were randomly divided into a training set (70%) and a test set (30%). Data from January to June 2024 were reserved as an external validation set. The study protocol was approved by the Research Ethics Committee of Qinghai Provincial People's Hospital (reference number: 2025-022-02), and informed consent was obtained from all participants or their legal guardians.

Inclusion criteria were as follows: (1) age ≥18 years; (2) documented history of hypertension; (3) cerebral hemorrhage confirmed by head CT and/or MRI. Exclusion criteria included: (1) traumatic cerebral hemorrhage, cerebral amyloid angiopathy, or secondary hemorrhage (e.g., aneurysms, vascular malformations, vasculitis, coagulopathies, tumor-related strokes, cerebral venous thrombosis, and so on); (2) incomplete clinical data insufficient for analysis; (3) loss to follow-up; (4) comorbidities that could confound study outcomes, such as life-threatening systemic diseases.

### 2.2 Predictor variables

This study defined poor prognosis as the failure of HICH patients to achieve expected clinical recovery goals 6 months post-onset. Detailed admission clinical data were retrospectively collected, including age, gender, hypertension history, diabetes history, smoking and alcohol consumption status, admission blood pressure, admission CT hematoma volume, admission blood glucose, surgical intervention, and admission Glasgow Coma Scale (GCS) score (15: conscious; 12–14: mildly impaired consciousness; 9–11: moderately impaired consciousness; 3–8: coma). Additionally, modified Rankin Scale (mRS) scores were recorded 6 months post-onset (0–2: good prognosis; 3–6: poor prognosis).

Imaging data included hemorrhage location (basal ganglia, thalamus, cerebellum, or lobar), initial hematoma volume (measured within 24 h of onset using open-source software 3DSlicer for layer-by-layer delineation), and ventricular rupture status. Laboratory data encompassed red blood cell count, hemoglobin, white blood cell count, platelet count, prothrombin time, international normalized ratio, activated partial thromboplastin time, fibrinogen, serum potassium, serum calcium, serum sodium, serum albumin, alanine aminotransferase, and aspartate aminotransferase.

### 2.3 Data pre-processing

Missing values frequently occur in medical datasets, which can impair model performance. To address this issue, multiple imputation was employed to handle missing data (10). Specifically, the Multivariate Imputation by Chained Equations (MICE) algorithm was utilized for this purpose. Additionally, continuous variables underwent standardization, and categorical variables were factorized. To mitigate class imbalance, the Random Over-Sampling Examples (ROSE) method was applied. In this study, we utilized the ROSE package in R to implement the algorithm. We followed the default settings of the package, which automatically determine the appropriate sampling ratio based on the imbalance of the dataset. This approach helps to improve the model's ability to generalize and make accurate predictions for both majority and minority classes (11).

### 2.4 Selection of candidate variables and predictors

For feature selection, Recursive Feature Elimination (RFE) was employed to identify the optimal subset of predictors. RFE, a widely utilized feature selection method in machine learning, enhances model accuracy and generalizability by eliminating redundant or irrelevant features. This process also reduces computational complexity and improves model interpretability. RFE was strictly conducted on the training dataset alone to avoid any potential information leakage. And during the RFE process, five-fold cross-validation was employed to ensure robustness and prevent overfitting. RFE analysis generated 25 potential predictors, from which the top 10 were selected for model development. The optimal feature subset included hematoma volume, GCS score, white blood cell (WBC) count, age, serum albumin, systolic blood pressure (SBP), blood glucose, platelet count, mean corpuscular volume (MCV), and serum potassium.

### 2.5 Machine learning models

We employed five machine learning models for training and validation:

- **SVM**: A supervised learning algorithm widely used for classification and regression tasks. SVM constructs hyperplanes to maximize the margin between classes, enabling effective data separation.
- **LR**: A generalized linear model commonly applied to classification problems. Its simplicity and interpretability make it a foundational tool in predictive modeling.
- **RF**: An ensemble learning method that constructs multiple decision trees to improve prediction accuracy and stability. RF's inherent resistance to overfitting and ability to handle high-dimensional data make it suitable for complex datasets.
- **KNN**: A straightforward yet effective algorithm used for classification and regression. KNN predicts outcomes by measuring distances between data points, with performance enhanced through feature selection and optimal *K*-value tuning.
- **XGBoost**: A state-of-the-art gradient-boosting framework that combines weak learners (typically decision trees) into a strong predictive model. XGBoost is renowned for its high performance, scalability, and support for diverse loss functions and regularization techniques.

Each model was selected based on its unique strengths in addressing classification tasks and handling complex clinical datasets.

### 2.6 Machine learning explainable tool

The model interpretation was performed using the SHAP method, which quantifies the contribution of each feature to the final prediction. By isolating independent feature contributions and analyzing feature interactions, SHAP provides a comprehensive and interpretable framework. Each observation in the dataset is associated with a unique set of SHAP values, enabling granular insights into individual predictions.

### 2.7 Statistical analysis

All statistical modeling and visualization analyses were performed using R software (version 4.4.2). Categorical variables were analyzed via chi-square tests or Fisher's exact probability method and reported as frequency percentages. Continuous variables following a normal distribution were described using mean ± standard deviation, with group comparisons conducted via *t*-tests. Non-normally distributed data were expressed as quartiles and analyzed for variability using the Wilcoxon rank-sum test. A significance level of (*P* < 0.05) was adopted.

The model's discriminative ability was quantified by the area under the receiver operating characteristic curve (AUC), complemented by assessments of sensitivity and accuracy. To evaluate clinical applicability, DCA was employed to calculate net benefit values across different risk thresholds, thereby assessing the decision-making utility of the predictive model.

## 3 Results

### 3.1 Patient characteristics

From June 2020 to June 2024, the Department of Neurosurgery at Qinghai Provincial People's Hospital admitted a total of 1,407 HICH cases. After applying the inclusion and exclusion criteria, 807 patients were included in the final cohort of this study. The data was divided into a training set (*n* = 497), a test set (*n* = 219), and an external validation set (*n* = 91). In the above-mentioned dataset, the proportion of missing values was 8.7%, and multiple imputation was performed. Baseline characteristic comparisons (Table 1) revealed statistically significant differences in Platelet Count (188.11 ± 64.70 vs. 172.01 ± 60.45, *P* = 0.001), APTT (26.37 ± 3.19 vs. 25.73 ± 3.25, *P* = 0.002), and WBC (10.67 ± 3.77 vs. 10.12 ± 3.83, *P* = 0.036), which were higher in the test set, while ALT levels were higher in the training set (28.63 ± 21.91 vs. 26.81 ± 21.41, *P* = 0.017).

**Table 1:** Demographic and clinical characteristics of the training and test set studies.

**Group**	**All data**	**Train data**	**Test data**	***P*-value**
N	817	497	219	
**Gender**				0.928
Female	203 (28.4%)	140 (28.2%)	63 (28.8%)	
Male	513 (71.6%)	357 (71.8%)	156 (71.2%)	
**Ethnicity**				0.458
Han	403 (56.3%)	280 (56.3%)	123 (56.2%)	
Tibetan	199 (27.8%)	133 (26.8%)	66 (30.1%)	
Hui	114 (15.9%)	84 (16.9%)	30 (13.7%)	
**Smoking**				0.556
Yes	619 (86.5%)	427 (85.9%)	192 (87.7%)	
No	97 (13.5%)	70 (14.1%)	27 (12.3%)	
**Drinking**				0.800
No	634 (88.5%)	441 (88.7%)	193 (88.1%)	
Yes	82 (11.5%)	56 (11.3%)	26 (11.9%)	
**Intraventricular hemorrhage**				0.515
No	393 (54.9%)	277 (55.7%)	116 (53.0%)	
Yes	323 (45.1%)	220 (44.3%)	103 (47.0%)	
**Bleeding location**				0.639
Basal ganglia	367 (51.3%)	261 (52.5%)	106 (48.4%)	
Thalamus	81 (11.3%)	52 (10.5%)	29 (13.2%)	
Cerebellum	80 (11.2%)	55 (11.1%)	25 (11.4%)	
Cerebral lobe	188 (26.3%)	129 (26.0%)	59 (26.9%)	
**Surgical treatment**				0.745
No	380 (53.1%)	266 (53.5%)	114 (52.1%)	
Yes	336 (46.9%)	231 (46.5%)	105 (47.9%)	
**Poor prognosis**				0.239
No	517 (72.2%)	352(70.8%)	165(75.3%)	
Yes	199 (27.8%)	145(29.2%)	54(24.7%)	
Age	58.28 ± 10.17	57.97 ± 10.28	58.98 ± 9.9	0.283
WBC	10.29 ± 3.82	10.12 ± 3.83	10.67 ± 3.77	0.036
RBC	5.39 ± 0.98	5.39 ± 0.97	5.38 ± 1.00	0.879
Hemoglobin	165.66 ± 29.90	165.88 ± 29.64	165.15 ± 30.54	0.852
MCV	90.97 ± 7.51	90.85 ± 7.89	91.25 ± 6.60	0.737
Platelet count	176.93 ± 62.18	172.01 ± 60.45	188.11 ± 64.70	0.001
PT	11.68 ± 1.10	11.65 ± 1.07	11.74 ± 1.16	0.233
INR	1.02 ± 0.15	1.02 ± 0.15	1.030 ± 0.15	0.343
APTT	25.93 ± 3.24	25.73 ± 3.25	26.37 ± 3.19	0.002
FBG	2.860 ± .90	2.860 ± .92	2.880 ± .87	0.648
ALT	28.07 ± 21.76	28.63 ± 21.91	26.81 ± 21.41	0.017
AST	26.44 ± 16.44	26.58 ± 16.41	26.11 ± 16.53	0.401
Albumin	38.24 ± 4.73	38.12 ± 4.76	38.53 ± 4.67	0.277
Blood glucose	7.52 ± 3.04	7.56 ± 3.05	7.45 ± 3.02	0.310
Serum potassium	3.69 ± 0.44	3.67 ± 0.45	3.72 ± 0.43	0.063
Serum sodium	137.53 ± 3.99	137.64 ± 3.90	137.29 ± 4.18	0.267
Serum calcium	2.26 ± 1.08	2.28 ± 1.29	2.22 ± 0.15	0.810
Body temperature	36.53 ± 1.17	36.51 ± 1.39	36.56 ± 0.30	0.963
Heart rate	79.79 ± 15.96	79.84 ± 15.77	79.67 ± 16.42	0.771
Respiratory rate	19.61 ± 1.86	19.63 ± 1.81	19.59 ± 1.97	0.446
SBP	163.47 ± 25.43	163.08 ± 25.61	164.35 ± 25.04	0.595
DBP	98.36 ± 15.62	98.41 ± 16.18	98.26 ± 14.29	0.834
Hematoma volume	24.05 ± 11.81	24.06 ± 11.87	24.03 ± 11.71	0.847
GCS	11.13 ± 2.62	11.11 ± 2.55	11.18 ± 2.75	0.624

Group comparisons were performed using the Wilcoxon rank sum test (continuous variables) and Fisher's exact test (categorical variables).

WBC, white blood cell count; RBC, red blood cell count; MCV, mean corpuscular volume; PT, prothrombin time; INR, international normalized ratio; APTT, activated partial thromboplastin time; FBG, fibrinogen; ALT, alanine aminotransferase; AST, aspartate aminotransferase; SBP, systolic blood pressure; DBP, diastolic blood pressure; GCS, glasgow coma scale; Hb, hemoglobin; PLT, platelet count; IVH, intraventricular hemorrhage.

Within the training set, comparisons between prognosis groups (Table 2) showed that the poor prognosis group exhibited significantly higher Hematoma Volume (30.64 ± 9.06 vs. 21.35 ± 11.84, *P* < 0.001) and WBC (11.20 ± 4.44 vs. 9.67 ± 3.46, *P* < 0.001), but lower GCS (9.85 ± 2.16 vs. 11.63 ± 2.53, *P* < 0.001). These findings highlight key clinical indicators associated with prognosis.

**Table 2:** Characteristics of HICH patients in the training set.

**Characteristic**	**Good prognosis**	**Poor prognosis**	***P*-value**
N	352	145	0.661
**Gender**			0.661
Female	97 (27.6%)	43 (29.7%)	
Male	255 (72.4%)	102 (70.3%)	
**Ethnicity**			0.063
Han	210 (59.7%)	70 (48.3%)	
Tibetan	87 (24.7%)	46 (31.7%)	
Hui	55 (15.6%)	29 (20.0%)	
**Smoking**			>0.999
No	302 (85.8%)	125 (86.2%)	
Yes	50 (14.2%)	20 (13.8%)	
**Drinking**			0.876
No	313 (88.9%)	128 (88.3%)	
Yes	39 (11.1%)	17 (11.7%)	
**Intraventricular hemorrhage**			0.013
No	209 (59.4%)	68 (46.9%)	
Yes	143 (40.6%)	77 (53.1%)	
**Bleeding location**			0.791
Basal ganglia	183 (52.0%)	78 (53.8%)	
Thalamus	40 (11.4%)	12 (8.3%)	
Cerebellum	38 (10.8%)	17 (11.7%)	
Cerebral lobe	91 (25.9%)	38 (26.2%)	
**Surgical treatment**			0.001
No	205 (58.2%)	61 (42.1%)	
Yes	147 (41.8%)	84 (57.9%)	
Age	57.10 ± 10.41	60.08 ± 9.68	0.008
WBC	9.67 ± 3.46	11.20 ± 4.44	< 0.001
RBC	5.38 ± 0.91	5.44 ± 1.11	0.788
Hemoglobin	165.79 ± 28.57	166.12 ± 32.20	0.754
MCV	90.70 ± 8.31	91.23 ± 6.76	0.874
Platelet count	172.17 ± 60.73	171.61 ± 59.97	0.910
PT	11.66 ± 1.02	11.62 ± 1.19	0.675
INR	1.02 ± 0.15	1.02 ± 0.16	0.973
APTT	25.75 ± 3.27	25.70 ± 3.21	0.828
FBG	2.84 ± 0.92	2.90 ± 0.90	0.579
ALT	29.85 ± 23.24	25.69 ± 18.03	0.170
AST	26.58 ± 16.58	26.59 ± 16.04	0.310
Albumin	38.07 ± 4.58	38.23 ± 5.18	0.579
Blood glucose	7.34 ± 3.05	8.08 ± 3.00	0.001
Serum potassium	3.68 ± 0.43	3.64 ± 0.49	0.312
Serum sodium	137.60 ± 3.54	137.72 ± 4.66	0.636
Serum calcium	2.22 ± 0.13	2.41 ± 2.39	0.330
Body temperature	36.56 ± 0.30	36.41 ± 2.54	0.513
Heart rate	79.73 ± 15.39	80.11 ± 16.72	0.865
Respiratory rate	19.47 ± 1.75	20.01 ± 1.89	0.023
SBP	160.24 ± 24.95	169.99 ± 25.96	< 0.001
DBP	97.45 ± 16.21	100.74 ± 15.94	0.080
Hematoma volume	21.35 ± 11.84	30.64 ± 9.06	< 0.001
GCS	11.63 ± 2.53	9.85 ± 2.16	< 0.001

WBC, white blood cell count; RBC, red blood cell count; MCV, mean corpuscular volume; PT, prothrombin time; INR, international normalized ratio; APTT, activated partial thromboplastin time; FBG, fibrinogen; ALT, alanine aminotransferase; AST, aspartate aminotransferase; SBP, systolic blood pressure; DBP, diastolic blood pressure; GCS, glasgow coma scale; Hb, hemoglobin; PLT, platelet count; IVH, intraventricular hemorrhage.

### 3.2 Model construction and evaluation

**Figure 1:**
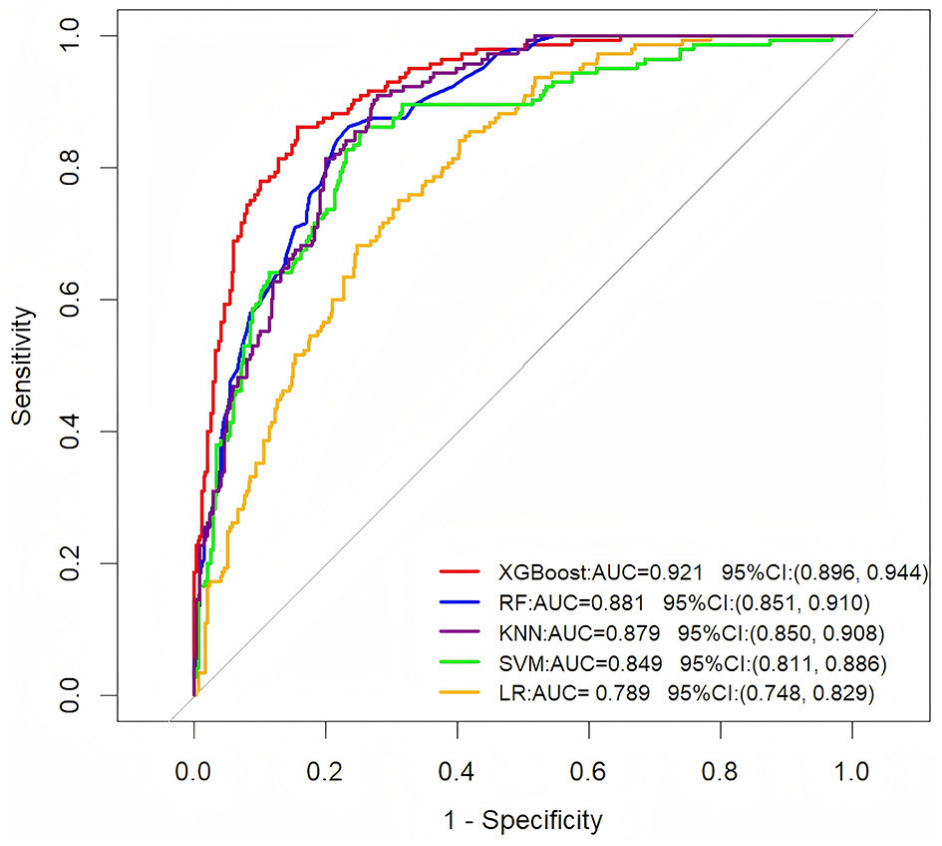
ROC curve analysis of five machine learning algorithms in the training dataset for predicting the long-term prognosis of HICH patients.

Using the training set data, we constructed five predictive models: XGBoost, RF, LR, SVM, and KNN. The training set was employed for training the models and performing hyperparameter tuning. We used a five-fold cross-validation approach on the training set to optimize model parameters during the development phase. The test set and the external validation set remained completely independent and were used only once after model selection and training were completed. This ensures an unbiased assessment of the model's generalization performance. The AUC values of the five ML models based on the training set are 0.921, 0.881, 0.789, 0.849, and 0.879, respectively (Figure 1). The XGBoost model demonstrated superior predictive accuracy, achieving an AUC of 0.921 (95% CI: 0.896–0.944), while the LR model showed relatively weaker performance (AUC = 0.789, 95% CI: 0.748–0.829).

**Figure 2:**
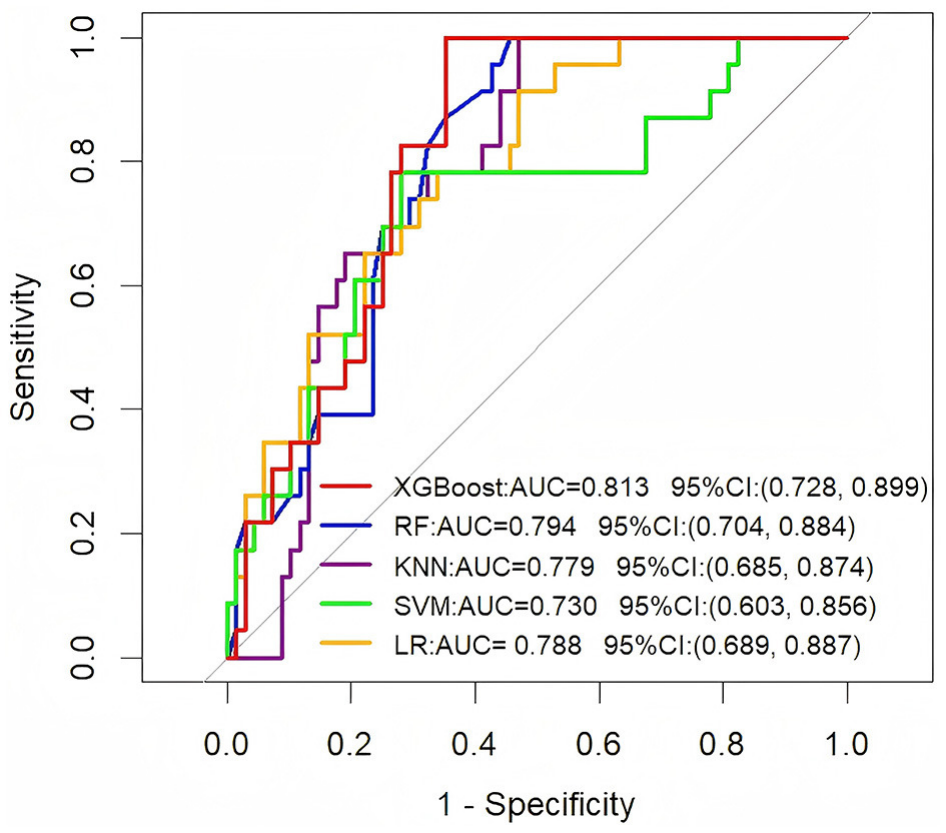
ROC curve analysis of five machine learning algorithms in the external validation set for predicting the long-term prognosis of HICH patients.

To further assess the generalization ability of these models, we evaluated their performance on an independent external validation set (Figure 2). The results showed that the XGBoost model sustained superior performance on the external validation set, achieving an AUC of 0.813 (95% CI: 0.728–0.899). This consistency with its training set performance indicates strong generalization capabilities. Other models also demonstrated varying levels of performance: the RF model achieved an AUC of 0.794 (95% CI: 0.704–0.884), the KNN model an AUC of 0.779 (95% CI: 0.685–0.874), the SVM model an AUC of 0.730 (95% CI: 0.603–0.856), and the LR model an AUC of 0.788 (95% CI: 0.689–0.887).

**Figure 3:**
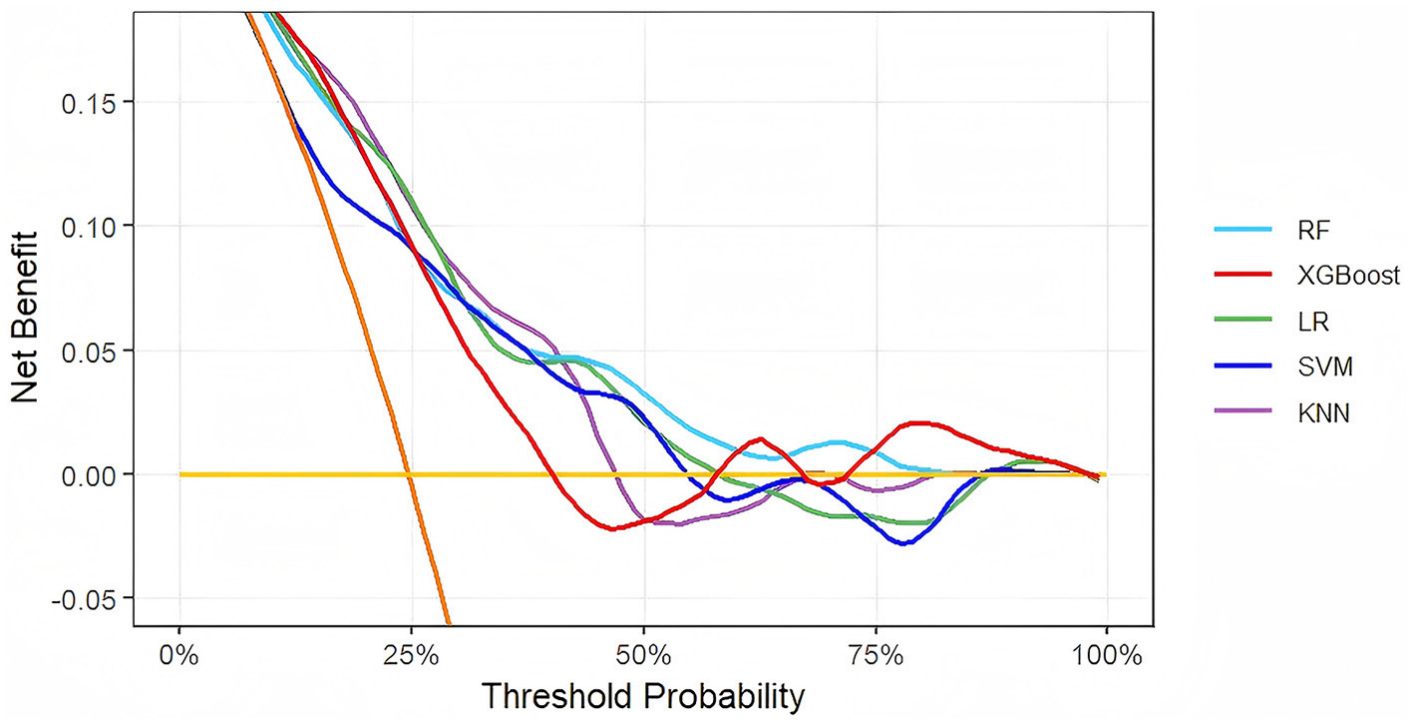
Decision curve analysis of five models plotting net benefits with different threshold probabilities.

To evaluate clinical utility, decision curve analysis (DCA) quantified the net clinical benefit of each model across threshold probabilities (Figure 3). All models outperformed the “treat all patients” (orange reference line) and “treat no patients” (yellow reference line) strategies. Notably, XGBoost provided the highest net benefit across a broad range of thresholds. Further performance assessment using metrics such as accuracy, sensitivity, positive predictive value (PPV), negative predictive value (NPV), and F1 score (Table 3) confirmed XGBoost's superiority. Consequently, XGBoost was selected as the core model for long-term HICH prognosis prediction.

**Table 3:** Predictive performance of the models.

**Model**	**AUC (%)**	**Sensitivity (%)**	**F1 score**	**Accuracy (%)**	**PPV**	**NPV**
XGBoost	0.921	0.634	0.715	0.853	0.821	0.862
RF	0.881	0.351	0.485	0.782	0.784	0.782
LR	0.789	0.386	0.466	0.742	0.589	0.778
SVM	0.849	0.379	0.516	0.792	0.808	0.790
KNN	0.879	0.324	0.456	0.774	0.770	0.775

AUC, area under the curve; PPV, positive predictive value; NPV, negative predictive value; XGBoost, extreme gradient boosting; RF, random forest; LR, logistic regression; SVM, support vector machine; KNN, K-nearest neighbors.

### 3.3 Interpretation of XGBoost model by SHAP method

**Figure 4:**
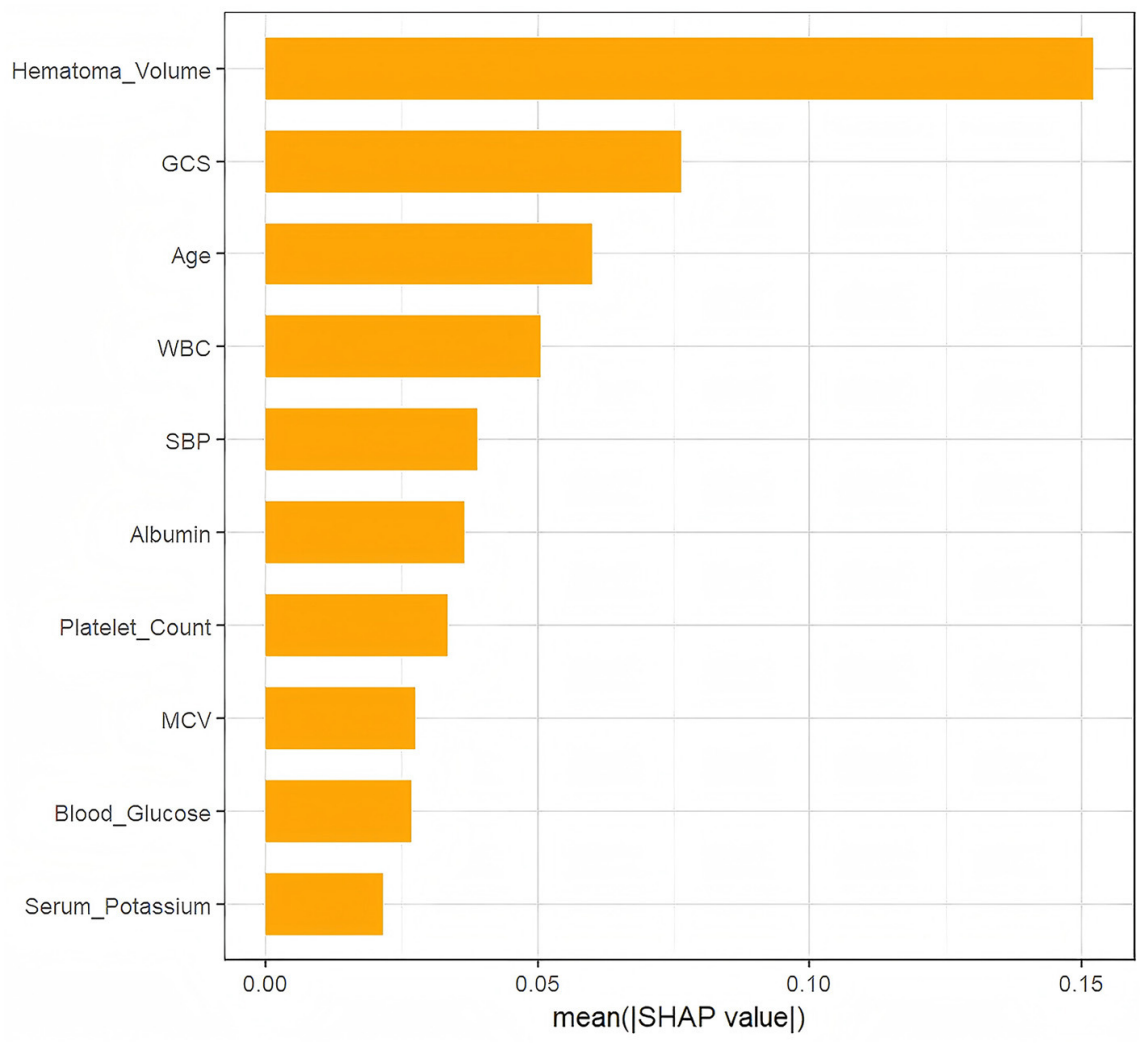
The weights of variables importance.

**Figure 5:**
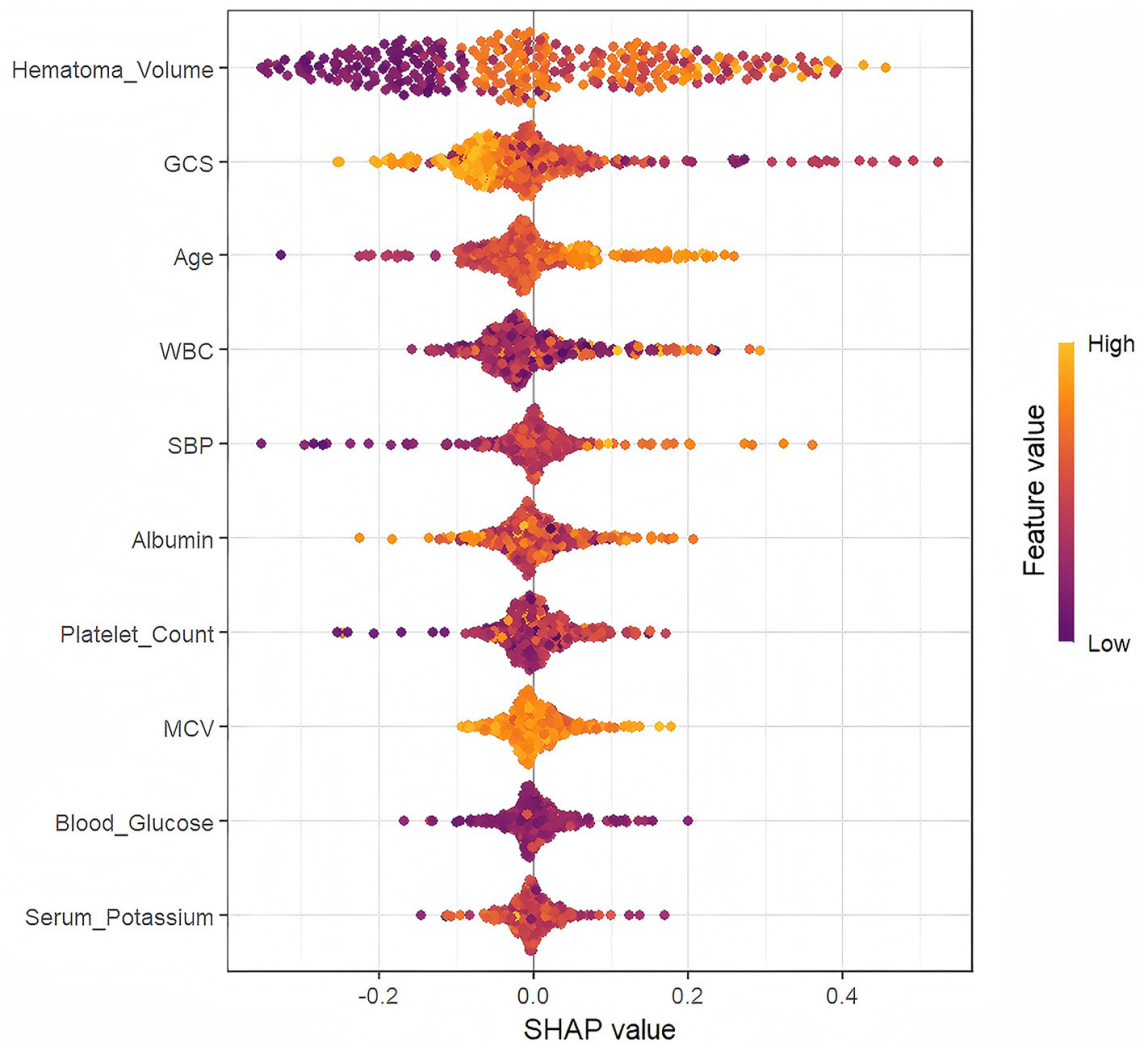
The SHapley Additive exPlanation (SHAP) values.

As depicted in Figure 4, Hematoma Volume emerged as the most influential predictor of prognosis, followed by GCS, WBC, Age, Albumin, and SBP. Figure 5 further elucidates the directional impact of each variable. Positive SHAP values (right side, orange) indicate features that increase the probability of poor prognosis, while negative values (left side, purple) suggest a reduced risk. Hematoma Volume showed a strong positive association with poor prognosis, with high values (orange) correlating with increased risk. Conversely, higher GCS scores (left side, orange) were linked to better outcomes, as indicated by negative SHAP values. For instance, larger hematoma volumes (right side, orange) were associated with poorer prognoses, whereas higher GCS scores (left side, orange) predicted better outcomes compared to lower scores (right side, purple).

### 3.4 SHAP individual force plots

**Figure 6:**
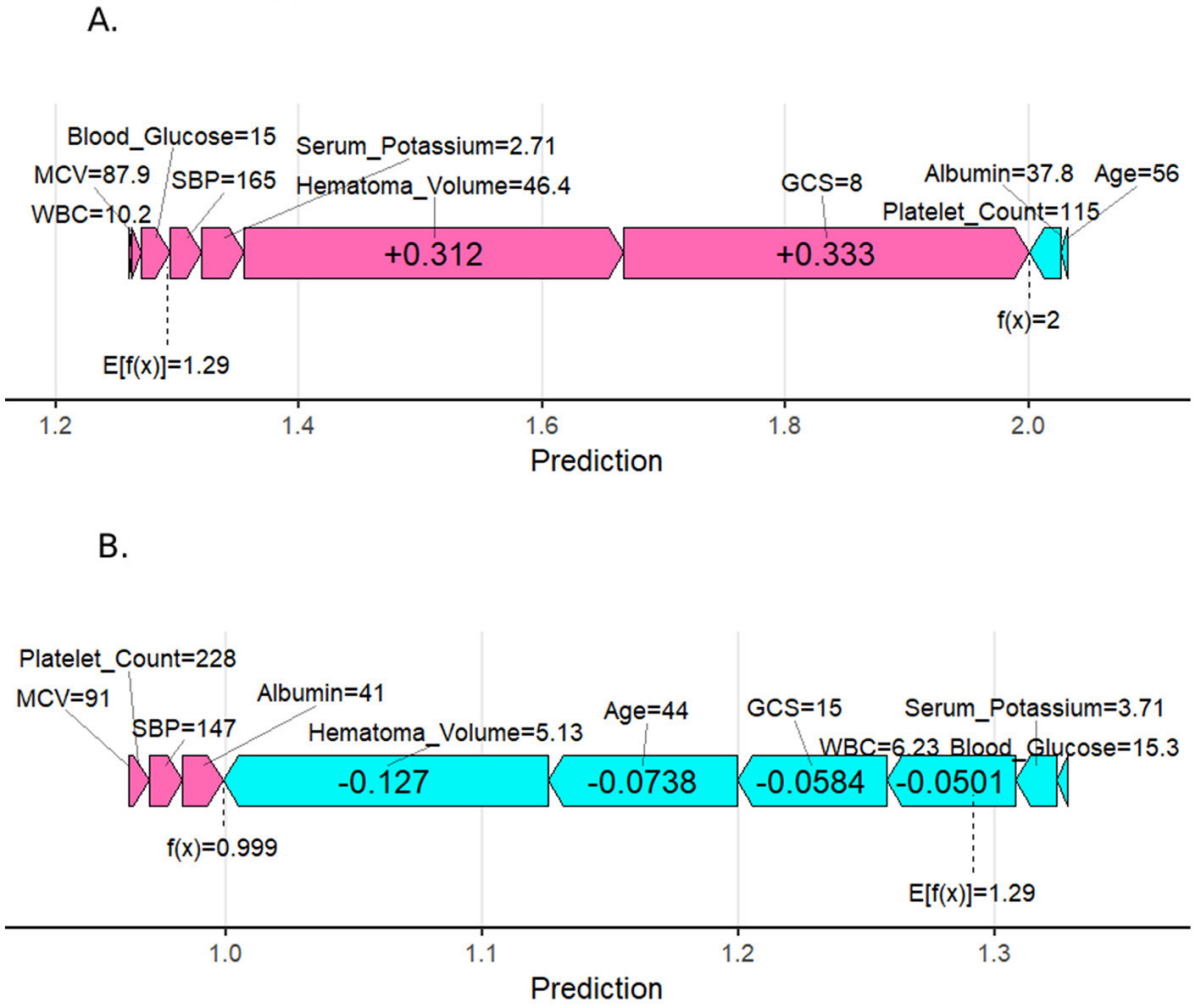
SHapley Additive exPlanation (SHAP) force plot for two selected patients. **(A)** Person with a poor prognosis. **(B)** Person with a good prognosis.

Figure 6 presents individual SHAP force diagrams for two patients: one with a poor prognosis (A) and one with a good prognosis (B). The model's base value (E[f(x)] = 1.29) represents the initial predicted value in the absence of feature inputs. The individual predictive value (f(x)) quantifies deviations from the base value using a logarithmic odds ratio, reflecting the cumulative effect of clinical characteristics on prognosis. In the diagram, red arrows denote risk-enhancing features, while blue arrows denote risk-suppressing features. The arrow length corresponds to the magnitude of the feature contribution. For Patient A (poor prognosis), high-risk features such as large hematoma volume (46.4 mL), low GCS score (8 scores), and metabolic abnormalities (e.g., blood glucose 15 mmol/L) collectively elevated the predictive value (f(x) = 2.0) above the baseline, strongly indicating adverse outcomes. Conversely, for Patient B (good prognosis), protective features dominated, driving the predictive value (f(x) = 0.999) below the baseline.

## 4 Discussion

Current research on HICH prognosis primarily focuses on identifying key prognostic factors and elucidating their mechanisms. While established early predictors include age, gender, smoking and alcohol history, neurological deficit severity, hematoma volume, intraventricular hemorrhage, and subarachnoid hemorrhage, their prognostic utility remains debated (4, 12, 13). Traditional approaches, such as univariate and multivariate logistic regression, have demonstrated limited accuracy in predicting outcomes. Machine learning algorithms, increasingly utilized in medical research (14), often outperform conventional statistical models. Recent applications in HICH include predicting hematoma expansion using techniques like XGBoost, which showed superior performance in early prognosis (15, 16). However, the use of machine learning for long-term functional recovery assessment remains underexplored.

In this study, we compared multiple machine learning algorithms and demonstrated, for the first time, the significant advantage of the XGBoost model in predicting 6-month HICH prognosis (AUC = 0.921 in the training set and AUC = 0.813 in the external validation set). The research findings confirm that the high performance of the XGBoost model reflects its genuine predictive capability, rather than overfitting. Sonobe et al. (17) constructed an RF model for predicting poor prognosis in ICH patients after rehabilitation therapy, and the model also demonstrated excellent performance. Previous studies have primarily employed machine learning algorithms to predict the short-term prognosis of HICH patients (18, 19). However, HICH patients possess the potential for continuous neurological recovery, and their neurological function may progressively improve over time. A longitudinal study conducted by Sreekrishnan et al. (20) on 173 HICH patients demonstrated that the mRS scores of most patients showed significant improvement at 3 and 6 months post-discharge. This study uniquely predicts the long-term prognosis of HICH patients through machine learning models. It can provide critical evidence for the development of personalized treatment and rehabilitation plans in clinical practice, thereby enhancing patient prognosis and quality of life. Using the SHAP method, we systematically evaluated the clinical weights of predictor variables, ranking them by importance. The SHAP individual force plot reveals the specific contributions of key pathophysiological indicators to the model predictions for individual patients. This holds significant value in enhancing the transparency of the model. Top-ranked variables, including hematoma volume and GCS score, were analyzed in conjunction with clinical insights, providing a foundation for individualized risk assessment and clinical decision-making.

Hematoma volume was identified as the most critical predictive variable in this study, aligning with previous findings (21). Larger volumes increase brain tissue compression, exacerbate blood-brain barrier disruption, and induce cerebral edema and intracranial pressure elevation, ultimately worsening neurological deficits (22). A study by Delcourt et al. (23) demonstrated that each 1 mL increase in hematoma volume raised the risk of death or dependence by 5%. Different brain regions exhibit significant threshold differences in their tolerance to hematoma volume due to variations in anatomical structure, functional importance, and compensatory capacity (24). Future research needs to further integrate location-specific volume thresholds to optimize prognostic scoring systems and intervention protocols.

Since its introduction in 1974, the GCS has become the international standard for assessing consciousness impairment in patients with traumatic brain injury and spontaneous cerebral hemorrhage (25). It indirectly reflects the extent of brain tissue damage (26). While GCS is widely used for acute-phase severity assessment, therapeutic decision-making, and long-term prognosis prediction, its predictive accuracy can be enhanced by integrating it with multidimensional indicators such as hematoma volume and age (27). Age emerged as another critical predictor (28), with advancing age significantly increasing the risk of adverse outcomes due to reduced physiological reserve and recovery capacity. A study by Huang et al. (29) highlighted aging as a key risk factor for poor prognosis in HICH.

Elevated peripheral blood WBC counts are a critical prognostic factor in HICH. A study indicates that an early increase in WBC levels after hemorrhage is closely associated with a higher mortality rate (30). This link may stem from inflammation triggered by brain tissue damage, which releases mediators attracting WBCs, primarily neutrophils and monocytes. Neutrophils, the first responders, phagocytose debris but also release reactive oxygen species (ROS) and matrix metalloproteinases (MMPs) like MMP-9. While these degrade damaged tissue, excessive ROS and MMPs can disrupt the blood-brain barrier, worsen cerebral edema, and cause secondary injury (31). High WBC levels often indicate increased risks of complications such as cerebral edema and rebleeding (32), which may lead to poorer long-term functional outcomes (33). However, a study by Morotti et al. (34) highlighted differing roles of neutrophils and monocytes: reduced neutrophil counts were tied to a higher risk of hematoma expansion, while elevated monocyte counts correlated with increased expansion risk. These findings underscore the complex relationship between leukocyte subsets and HICH prognosis, suggesting a need for further research into the specific mechanisms of WBC action post-hemorrhage.

Serum albumin levels exhibit a significant negative correlation with poor prognosis in HICH. Research consistently indicates that low serum albumin is strongly associated with increased mortality and adverse outcomes in HICH patients. Specifically, diminished albumin levels may reflect severe comorbidities (Acute inflammation, infection, liver disease, or vascular endothelial injury) or malnutrition, both of which contribute to poor prognoses. Studies have highlighted that reduced albumin levels directly correlate with higher mortality risk (35). Furthermore, admission albumin levels serve as an independent prognostic indicator. One study observed that patients with low admission albumin had prolonged hospital stays and elevated short-term and long-term mortality rates (36). In clinical practice, monitoring albumin levels provides critical insights into patient prognosis. Physicians should closely track these levels and consider interventions to enhance nutritional status or address underlying conditions.

Systolic blood pressure (SBP) is a key risk factor in hypertensive HICH, impacting severity and prognosis. Each 10 mmHg SBP increase raises hemorrhage risk by 60% (37). In the acute phase, high SBP exacerbates hemorrhage and may cause further brain damage by increasing cerebral blood flow and intracranial pressure (38). Therefore, stabilizing SBP and minimizing fluctuations are essential for improving long-term prognosis. Clinical guidelines recommend controlling acute-phase SBP to approximately 140 mmHg, as this level is associated with reduced poor prognosis risk (39, 40). However, overly rapid SBP reduction may adversely affect short-term and long-term outcomes (41). Rational SBP management can significantly lower the risk of poor prognosis and enhance patients' quality of life.

## 5 Conclusion

We developed an interpretable XGBoost prediction model that demonstrated superior performance in assessing the risk of poor prognosis in patients with HICH. Furthermore, by quantifying the specific contributions of key pathophysiological indicators to individual patient model predictions through the SHAP framework, individualized risk stratification and optimization of medical resource allocation can be achieved.

## 6 Strengths

This study's strength lies in constructing a long-term prognostic prediction model for the high-risk HICH subtype (comprising 50%−70% of spontaneous cerebral hemorrhage cases), addressing the etiological heterogeneity limitations of broader sICH models. Beyond traditional indicators like hematoma volume and GCS score, the study confirmed the independent predictive value of serum albumin, white blood cell count, and systolic blood pressure fluctuations for HICH's long-term prognosis. The model's real-world generalizability is supported by external validation and decision curve analysis across independent time periods. Clinicians can utilize this model to identify at-risk patients and optimize rehabilitation resource allocation. Additionally, the SHAP framework's application enhances model transparency, offering an interpretable basis for personalized interventions.

## 7 Limitations

Our study has several limitations. First, the GCS is influenced by patient cooperation and rater experience, which may introduce data bias. Second, as a retrospective analysis, selection bias may affect the generalizability of the results. Third, the limited number of externally validated cases may impact the reliability of the findings. Fourth, since the data is sourced from a single institution, there are limitations in terms of coverage and diversity, which may result in the analysis lacking comprehensiveness and broad representativeness. Finally, future research should not only focus on developing high-performance predictive models but also aim to create accessible application platforms.

## Data Availability

The raw data supporting the conclusions of this article will be made available by the authors, without undue reservation.
